# Gender differences in medical errors among older patients and inequalities in medical compensation compared with younger adults

**DOI:** 10.3389/fpubh.2022.883822

**Published:** 2022-09-20

**Authors:** Paicheng Liu, Yuxuan Yang, Jianxin Cheng

**Affiliations:** ^1^School of Public Administration, Southwestern University of Finance and Economics, Chengdu, China; ^2^School of Government, Sun Yat-sen University, Guangzhou, China; ^3^School of Public Administration and Emergency Management, Jinan University, Guangzhou, China

**Keywords:** medical errors, older patients, medical compensation, health inequalities, gender differences

## Abstract

**Background:**

Despite growing evidence focusing on health inequalities in older adults, inequalities in medical compensation compared with younger adults and gender disparities of medical errors among older patients have received little attention. This study aimed to disclose the aforementioned inequalities and examine the disparities in medical errors among older patients.

**Methods:**

First, available litigation documents were searched on “China Judgment Online” using keywords including medical errors. Second, we compiled a database with 5,072 disputes. After using systematic random sampling to retain half of the data, we removed 549 unrelated cases. According to the age, we identified 424 and 1,563 cases related to older and younger patients, respectively. Then, we hired two frontline physicians to review the documents and independently judge the medical errors and specialties involved. A third physician further considered the divergent results. Finally, we compared the medical compensation between older and younger groups and medical errors and specialties among older patients.

**Results:**

Older patients experienced different medical errors in divergent specialties. The medical error rate of male older patients was over 4% higher than that of females in the departments of general surgery and emergency. Female older patients were prone to adverse events in respiratory medicine departments and primary care institutes. The incidence of insufficient implementation of consent obligation among male older patients was 5.18% higher than that of females. However, females were more likely to suffer adverse events at the stages of diagnosis, therapy, and surgical operation. The total amount of medical compensation obtained by younger patients was 41.47% higher than that of older patients.

**Conclusions:**

Except for the common medical errors and departments involved, additional attention should be paid to older patients of different genders according to the incidence of medical errors. Setting up the department of geriatrics or specialist hospitals is also an important alternative to improve patient safety for older people. Furthermore, there may be inequality in medical compensation in older patients due to the tort liability law of China.

## Introduction

It is estimated that by 2050, one-fifth of the population in poor countries will be over 60 years old, and the rate of aging has been increasing particularly in developing countries (1). However, older patients may be more susceptible to the double harm caused by medical service errors and insufficient medical compensation, which aggravates the inequality and emphasizes the relative disadvantage of older adults in terms of health and quality of life.

Most studies on patient safety have suggested that older patients experience a higher incidence of medical errors than other patient groups (2–4). Justiniani (5) and Steel et al. (6) initially recorded the occurrence of medical errors related to older people, and subsequent studies have emphasized that older patients are prone to be the victims (2, 3). Furthermore, medical errors have been posing a great threat to patient safety and have become a stumbling block to improving the quality of medical services (7).

However, the compensation arising from medical errors and accompanying disputes is allegedly unjust (8). Medical errors could cause over 20% of physician-patient disputes (9), and induce several litigations (10, 11). In China, medical disputes between physicians and patients have intensified and reached unprecedented status (12), but the compensation for different medical damages is unfair (13). The inequalities in medical errors and compensation among different groups of patients have been minimally evaluated. Developing countries such as China are accelerating their entry into an advanced stage of aging. This will enlarge the demand for medical services by older patients (14), and make it urgent to unravel the origins of the disparity in medical errors and compensation.

Existing studies on patient safety for older people have mainly focused on medication errors and reasons for medical errors (15, 16). The Harvard Medical Practice Study (HMPS) demonstrated that patients aged over 65 years occupied 27% of hospitalized population, but accounted for 43% of all adverse events (2). Another retrospective study conducted in Portugal revealed more serious consequences, with patients aged over 65 years accounting for 59.2% of all patients who experienced adverse events (17). First, the incidence of medical errors might rise significantly with age (3). Compared with patients aged 16–44 years, patients aged 65 years or more have a two-fold higher risk of adverse events (18). Second, in addition to the age factor, the complexity, comorbidity, reduced functional ability, disease severity, and lower quality of care are also important reasons for the higher scale of medical errors among older individuals (19, 20). A recent study has found that adverse event rates and outcomes in older patients are related to the complexity and frailty rather than solely to age and comorbidity (21). Besides, systematic reviews have found that over- and under-diagnosis are relatively more common among older patients (19, 22). Third, the higher incidence of medical errors among older patients derives from the more frequent medical visits compared with other groups (23). A previous study found that people over the age of 65 years visited their doctor on an average of eight times per year, compared with the general population's average of five visits per year (24).

There are still some gaps in the knowledge about the disparity of the medical harm suffered by older patients. First, while most studies have inclined to regard old patients as a whole, it would be of interest to examine whether there is any difference in the distribution of medical errors among different departments of the hospitals. Moreover, it would be useful to understand the variations among different subgroups of older patients, such as subgroups based on gender differences; this would reveal the intragroup variations that contribute to the older patients' risk of medical errors. Second, it should be investigated whether fair compensations have been obtained by older patients for medical errors. Medical errors could exacerbate the patients' situations and increase their medical expenses (25), so the fairness of compensation is relevant to the total equality or inequality of patient safety.

To clarify the abovementioned aspects, we utilized the available dataset from “China Judgments Online”, the most comprehensive lawsuit website in China, to compare the medical compensation between older patients and other groups. We calculated the average total compensation for older and younger patients and compared the average compensation for each item to disclose the health inequalities between them. Moreover, our second objective was to investigate the gender differences in medical errors among older patients according to the medical litigation documents. We summarized the types of medical errors among the older patients and medical departments involved, from the perspective of different genders. Based on the results of this study, we put forward countermeasures and recommendations for older patients of different genders.

There are several practical implications of this study. First, we consider that concerning the patient safety of the elderly group is the prerequisite for ensuring the effectiveness of clinical medical services. Despite medical errors in older patients having been extensively investigated, empirical analysis from a gender perspective is still deficient. Second, litigation documents betrayed medical errors from a different side. Litigation analysis has been confirmed to enhance clinical risk management for physicians in Italy (26), and Chinese physicians could benefit from the conclusion of this study as well. Third, we aim to disclose the inequalities in medical compensation due to medical errors between younger and older patients to achieve a more rational and fairer scheme of medical claims.

## Methods

### Study design and samples

“China Judgments Online” delivers a large number of accessible data for the research of medical errors among older patients. According to the provisions of the Supreme People's Court of the People's Republic of China, except for particular circumstances stipulated by law, legally effective judgments, rulings, and decisions should generally be available on the Internet. Therefore, we retrieved litigation documents related to medical errors on the public website for research without the need for ethical approval. “China Judgment Online” is currently the largest website for litigation documents in the world. More importantly, these litigation documents introduce detailed patient information and adverse events that they suffered. Especially, the items and amounts of compensation obtained by patients provided data support for our study.

First, we utilized a computer crawler to accomplish a comprehensive search of medical litigation cases according to the keywords including medical errors and retrieved 5,072 judgment documents related to medical disputes. Second, we extracted half of these using a systematic random sampling method and retained 2,536 cases. Third, we deleted irrelevant cases in the database through manual reading. In this stage, 549 cases were not associated with medical disputes or the patient (plaintiff) claims of medical errors were not confirmed by the court. Finally, we invited three frontline physicians to determine the medical errors and departments involved. We also coded the medical compensation amount and specific items that patients obtained based on the content of litigation documents.

### Data collection

We consulted existing research on the classification of medical errors and clinical departments (27). The table of medical error classification is available in Appendix. To guarantee the reliability and validity of this study, we first sent the medical litigation documents to two physicians (one physician is from the Department of Gastroenterology, and the other is from the Department of Radiotherapy) and asked them to independently judge the medical errors and departments involved. Then, we invited a third physician (from the Department of Orthopedics) to review the divergent results and make a final decision. The three frontline doctors had more than 7 years of clinical experience and had completed training in all medical departments. Their medical skills were professional, and their judgments were also relatively objective, fair, and effective.

**Figure 1:**
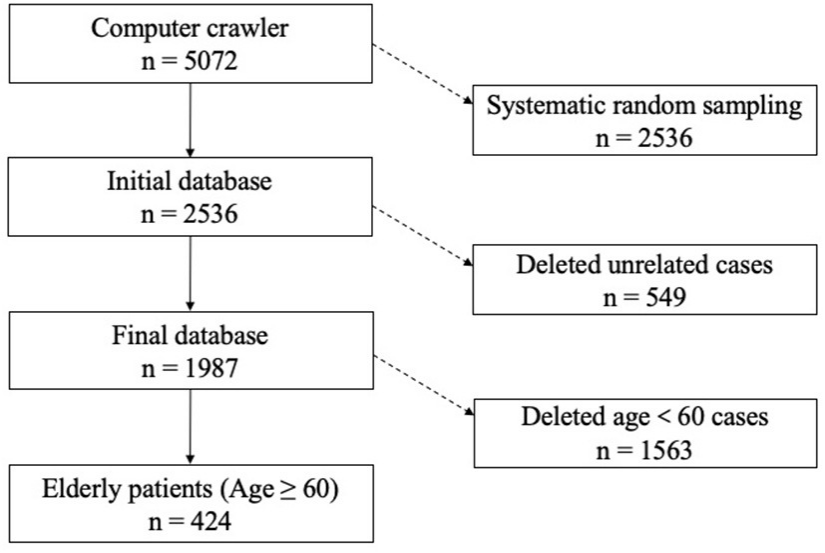
Process of the data collection.

In addition, according to the international definition for older patients, we classified patients over 60 years old (including 60 years old) as older patients based on the plaintiff (patient) information mentioned in the litigation documents and obtained a sample of 424 cases. The remaining 1,563 cases under 60 years old were regarded as younger patients. We aimed to analyze the differences in medical compensation between these two groups of patients. Data analysis was done in Stata 16.0. Figure 1 illustrates the entire process of data collection. The aforementioned medical litigation documents are legal after the court's judgment takes effect. More importantly, the contents of these cases are based on objective facts from both physicians and patients regarding clinical interactions and are representative of the errors that occur in practical circumstances.

## Results

### Older patients may suffer medical errors in different medical departments

Orthopedics, general surgery, and cardiology were the three medical departments where older patients suffered most of the medical errors. The proportion of medical errors in the orthopedics department for male older patients was the same as that for females. However, in the general surgery department, the medical error rate of male older patients was above 4% higher than that of females (Table 1). The difference in the emergency department was also obvious, and the incidence of medical errors among male older patients was nearly twice as high as that among females. Overall, the incidence of medical errors of male older patients was 6.37% higher than that of females in surgical departments.

**Table 1:** Proportion of medical errors among older patients in each department.

**Male**			**Female**		
**Specialty**	**Subspecialty**	**Percent**	**Specialty**	**Subspecialty**	**Percent**
Internal medical departments	Cardiology	10.28%	Internal medical departments	Cardiology	9.82%
(25.69%)	Neurology	4.67%	(27.6%)	Neurology	6.75%
	Respiratory medicine	3.27%		Respiratory medicine	6.13%
	Gastroenterology	3.27%		Nephrology	2.45%
	Nephrology	1.4%		Gastroenterology	1.84%
	Hematopathology	1.4%		Rheumatology and immunology	0.61%
	Endocrinology	0.93%			
	Rheumatology and immunology	0.47%			
Surgical departments	Orthopedics	17.29%	Surgical departments	Orthopedics	17.18%
(49.07%)	General surgery	17.29%	(42.34%)	General surgery	13.5%
	Neurosurgery	5.61%		Cardiothoracic surgery	4.91%
	Cardiothoracic surgery	4.67%			
	Urinary surgery	3.74%		Neurosurgery	3.68%
	Vascular surgery	0.47%		Urinary surgery	3.07%
Specialist departments	Emergency department	8.41%	Specialist departments	Emergency department	4.29%
(18.68%)	Ophthalmology	2.34%	(16.57%)	Ophthalmology	3.07%
	Oncology	1.87%		Oncology	3.07%
	ICU	1.4%		E.N.T.	1.23%
	Dermatology	1.4%		Dermatology	1.23%
	Stomatology	1.4%		Psychiatry	1.23%
	Psychiatry	0.93%		Obstetrics-gynecology	1.23%
	E.N.T.	0.93%		ICU	0.61%
				Stomatology	0.61%
Medical technology departments	Anesthesiology	0.47%	Medical technology departments	Anesthesiology	1.84%
(0.47%)			(3.07%)	Blood transfusion	1.23%
Primary care institutes		6.09%	Primary care institutes		10.42%

ENT, Department of Ear, Nose, and Throat; ICU, Intensive Care Unit.

Among female older patients, except for the internal medicine departments, the medical error proportions in the surgical, specialist, and medical technology departments were all lower than those among male older patients. Although the proportion of medical errors in the internal medicine departments among female older patients was higher than that among males, there were fewer types of subspecialties involved. Particular attention should be paid to female older patients in the nephrology, respiratory, and oncology departments. The incidence rates of medical errors in these three departments were higher in females than in males. The rate of female older patients suffering medical errors in primary care institutes was nearly 1.7 times higher than that of males, which is the department with the third-highest number of medical errors for female older patients besides orthopedics and general surgery.

### There are significant gender variations in medical errors among older patients

**Figure 2:**
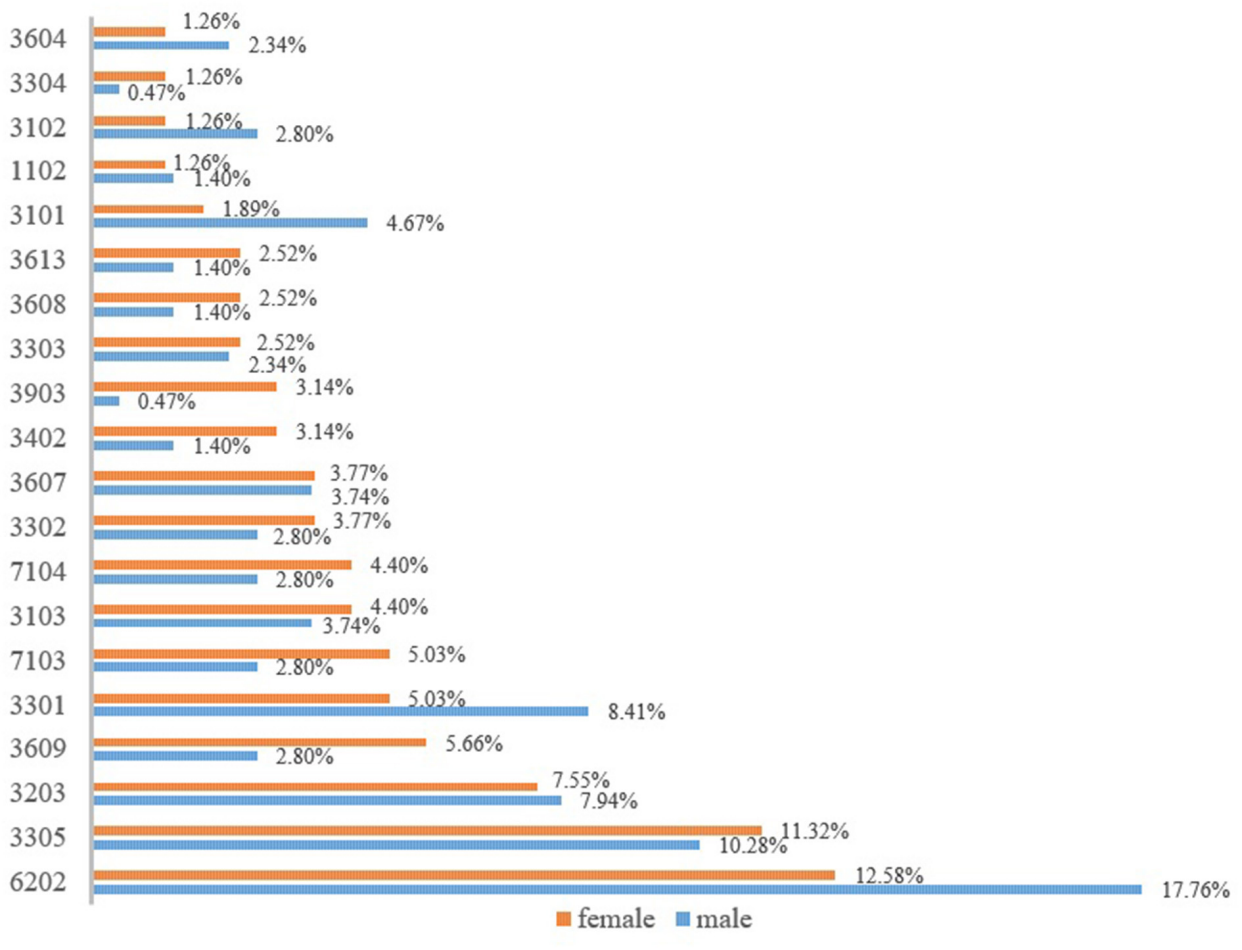
Major medical errors among older patients according to gender.

To better compare the disparity of medical errors between male and female older patients, we selected the top 20 medical errors with the highest frequencies for comparative analysis. Figure 2 shows that in addition to the near incidence of male and female older patients with insufficient inspection (3,203), failure to cure protopathy (3,607), treatment error (3,303), and medical staff not qualified to practice medicine (1,102), there were remarkable differences in other medical errors between the two subgroups (The codes for medical errors can be found in Appendix). We found that the medication errors were rare among older patients. Instead, invalid communication led to more insufficient implementation of consent obligation (6,202) among older patients.

Male older patients were more prone to insufficient implementation of consent obligation than females. The incidence of this medical error among male older patients was 5% higher than that among females. Meanwhile, the delay of timing during treatment (3,301) and misdiagnosis (3,101) were also more frequent among male older patients. For the remaining medical errors, involving the stages of therapy, surgical operation, and diagnosis, the incidence among female older patients was higher than that among male patients. It is worth noting that female older patients also had a higher incidence of errors in medical record writing than males, including informal writing or modification of medical records (7,103) and incomplete medical records (7,104). Although there were still some other medical errors that occurred frequently among female older patients, their proportion was not high.

### Older patients obtain less medical compensation than younger patients

We revealed the inequities in medical compensation between the two groups by calculating the mean compensation amount for older patients and younger patients. To further compare the disparity of medical compensation between older and younger patients, we also calculated the mean compensation amount for each item. As shown in Table 2, younger patients obtained higher total compensation than older patients. We calculated the ratio of the difference between the two compensations and the compensation for older patients. The total amount of compensation obtained by younger patients was 41.47% higher than that obtained by older patients. There was obvious inequality in compensation for medical errors between older and younger patients.

**Table 2:** Comparison of compensation between older and younger patients.

**Item**	**Younger patients**		**Older patients**		**(Age < 60 – age ≥ 60)/** **age ≥ 60**
	**Amount ($)**	***N*** **(%)**	**Amount ($)**	***N*** **(%)**	
Total compensation	31,270	1,302 (94.9%)	22,104	390 (96.3%)	41.47%
Total compensation	29,167	665 (48.91%)	21,012	288 (71.3%)	38.81%
(remove “work”)					
Mortuary	1,004	5 (0.36%)	169	1 (0.25%)	494.08%
Later treatment	23,339	90 (6.5%)	5,470	21 (5.15%)	326.67%
Recovery	2,119	8 (0.58%)	940	2 (0.49%)	125.43%
Work	2,103	637 (45.99%)	1,092	102 (25%)	92.58%
Death	29,490	385 (27.8%)	16,822	194 (47.55%)	75.31%
Alimony	5,908	243 (17.55%)	3,457	35 (8.58%)	70.90%
Accommodation	457	195 (14.08%)	276	36 (8.82%)	65.58%
Disability	35,630	581 (14%)	21,598	135 (11%)	64.97%
Nursing	6,440	801 (57.83%)	3,910	233 (57.11%)	64.71%
Post	13	10 (0.72%)	8	5 (1.23%)	62.50%
Others	3,862	59 (4.26%)	2,634	29 (7.11%)	46.62%
Medical	5,117	959 (69.24%)	3,739	261 (63.97%)	36.85%
Emotion	2,991	958 (69.17%)	2,843	310 (75.98%)	5.21%
Burying	1,501	379 (27.36%)	1,473	197 (48.28%)	1.90%
Traffic	256	865 (62.45%)	256	232 (56.86%)	0.00%
Print	28	47 (3.39%)	28	25 (6.13%)	0.00%
Food	350	762 (55.02%)	357	229 (56.13%)	−1.96%
Appraisal	701	584 (42.17%)	747	149 (36.52%)	−6.16%
Lawyer	950	52 (3.75%)	1,025	23 (5.64%)	−7.32%
Expert consulting	238	11 (0.79%)	271	3 (0.74%)	−12.18%
Nutrition	542	474 (34.22%)	736	120 (29.41%)	−26.36%
Examination	374	25 (1.81%)	535	5 (1.23%)	−30.09%
Insurance	1,321	5 (0.36%)	3,186	2 (0.49%)	−58.54%

Since most older patients exceeded the legal age for work, they could not obtain compensation items of work compared with younger patients. To control the interference of work items on the equality of medical compensation, we removed it and repeated the calculation. Regrettably, the mean total amount of compensation (after removing work) obtained by younger patients was still 38.81% higher than that obtained by older patients, which is only 2.66% lower than the previous result. We confirmed to some degree that older patients may have encountered health inequality in medical compensation after suffering from medical errors. In other words, there might be other specific medical compensation items that produced health inequality among older patients.

With regards to the other aspects of specific items, older patients' compensations were significantly lower in the mortuary, later treatment, recovery, death, alimony, and disability compensation. The disadvantages of older patients in the aforementioned compensations have resulted in a significantly lower total amount compared with younger patients. Particularly, younger patients received 494.08 and 326.67% more compensation than older patients in the mortuary and later treatment items, respectively. Thus, we may conclude that older patients are at a higher risk of medical errors but with less equality of medical compensation. As shown in Table 1, the proportion of older patients who died was ~20% higher than that of younger patients, but the amount of death compensation for the young was 75.31% higher than that for the older patients.

In summary, on the one hand, there are variations in the medical errors and departments involved within the older patient group. On the other hand, there is also inequality in the amount of medical compensation for older patients compared with younger patients. Generally speaking, older patients fail to acquire enough medical compensation, which may cause older patients to endure more severe health inequalities.

## Discussion

There are disparities in medical errors and involved specialties among older patients of different genders. Also, older patients involved in lawsuits due to medical errors obtain lower compensation compared with younger patients, which causes health inequalities for older patients. Despite growing evidence focusing on health inequalities among older patients, such as rural-urban differences (28) and socioeconomic factors (29, 30), the contribution of this study is to explore the gender disparities in medical errors among older patients and compare the inequalities in medical compensation.

### Insufficient implementation of informed consent obligations is worse in older patients

Existing studies have indicated that older patients have significantly weaker comprehension of informed consent information (31). Compared with younger patients, older patients could encounter more barriers when communicating or interacting with physicians (32). In this study, we also revealed that insufficient implementation of informed consent obligation was the most frequent medical error both among male and female older patients. Previous studies have shown that in the process of interaction between patients and physicians, older patients, particularly the very old and less well educated, were more likely to place physicians in a dominant role and themselves in a submissive role (33). Therefore, we consider that during the clinical reception with older patients, physicians should pay more attention to whether their words are completely comprehended by older patients, to avoid the medical errors related to communication.

### Be alert to the disparities in medical errors and departments among older patients

In terms of medical departments, we drew the same conclusion as previous studies. Orthopedics is the department with the most medical errors (34), regardless of whether it is for male or female older patients. However, we should be more careful with the male older patients in the general surgery and emergency departments, and with the female older patients in the department of respiratory medicine. Although adverse events during acute care are more frequent among older patients (35), we further found that male older patients in the emergency department were nearly twice as likely to experience medical errors as female patients. Because of the divergent incidence rates of medical errors between male and female older patients in these medical departments, it is necessary to pay different attention to the older patients depending on gender. Furthermore, we found that female older patients suffered more medical errors in primary care institutes, which may be related to the fact that female patients prefer primary care (36). Despite several years of primary care reform in China, current performance remains poor (37). The older population's concerns about patient safety may counteract the effects of easy availability in primary care institutes. Hence, an accountability framework for community health service is imperative in China.

### Setting the department of geriatrics or specialist hospitals

Due to complexity and comorbidity, a sole medical department could no longer meet the medical demands of older patients. We illustrated that the medical errors encountered by older patients covered many clinical stages, including diagnosis, therapy, and surgical operation. Moreover, in practice, older patients may suffer from multiple chronic diseases (38, 39), so treatment in a single medical department could no longer meet their health demands. Thus, transferring between different medical departments or clinical sites has become an inevitable choice for older patients.

However, frequent transfer of clinical sites may easily cause missed information and adverse events among older patients (40). Although some scholars suggested that physicians who provided medical services to older patients rarely experienced specialized training related to geriatrics (41), it is more vital to set up medical geriatric departments and even specialized hospitals for older patients (42, 43). China has made great strides in the accessibility and quality of health services (44). Yet, there have not been sufficient physicians in China to provide specialist clinical services for older patients so far. We suggest that in the phases of medical education, specialized curriculums on geriatrics should be supplemented, and hospital staff should receive more training about geriatrics (45). Additionally, the government can consider piloting geriatric specialties in public hospitals, which can not only improve patient safety but also result in accelerated efforts and control practice risk for physicians (46).

Analyzing the types of medical errors among older patients is beneficial for the aging population around the world. The data in this study come from China, a country encountering a promptly aging population, which poses a burden to its health care delivery and society at large. However, how to ensure the safety of older patients and provide higher-quality health services should be undoubtedly universal in an increasingly aging world. Importantly, satisfying the clinical service needs of older patients is an important approach to reducing health inequities.

### Laws related to medical claims are not beneficial for older patients

According to our results, it can be perceived that some items and the total amount of medical compensation (regardless of whether or not we removed the “work” item) for older patients are lower than those for younger patients. Because most of the older patients have retired or lost the ability to work in practice, they remarkably lack the compensation item of the work. Except for the medical appraisals in the judicial system of China (47), the total amount of compensation for older patients is lower, which may be due to the age limit for death and disability compensation in Chinese law. After referring to the Tort Liability Law of the People's Republic of China, we noticed that there are diverse rules for compensation between older and younger patients.

For patients over 60 years old, death and disability compensation in China are calculated 1 year less for every additional year of age. When patients under the age of 60 could obtain 20 years of compensation if suffering death or disability, patients over 60 years old could consequently obtain less compensation due to the higher age. The provision of the law directly leads to health inequality in medical compensation for older patients in China. This is also why the results of this study indicated that younger patients acquired more total compensation for medical claims.

As for younger patients obtaining more mortuary and later-treatment fees, this may be related to the possibility that they experience more severe adverse events (48). This study was limited by the data and could not measure the severity of adverse events encountered by different patients, but the proportion of younger patients among the claimants was indeed higher, and they were much more likely to obtain the indemnity as well, which is consistent with the findings of existing studies (49). Additionally, older people might have disadvantages in the ease of access to legal support because their adult children usually perform the plaintiff instead, which can be confirmed by the litigation documents. Regarding the health inequality in compensation for older patients, we believe that a legal litigation aid system should be built to strengthen older patient protection and safety. Every hospital should establish a medico-legal watchdog responsible for collecting and analyzing information on professional medical liability disputes in order to prevent and manage such events as well (50).

## Conclusions

We found that there were disparities in medical errors and specialties between male and female older patients. In addition to the department of orthopedics, where medical errors are the most common for older patients, particular attention should be paid to male older patients in the emergency department and female older patients in the respiratory medicine department. Female older patients suffered adverse events in the stages of diagnosis, therapy, and surgical operation, while male older patients were prone to harmful effects by the physicians in insufficient implementation of consent obligation. Furthermore, older patients obtained more unreasonable medical compensation due to the current provisions of the Tort Liability Law of the People's Republic of China, which also led to health inequality compared with younger patients. It is necessary to build a legal litigation aid system to enhance patient safety and clinical protection for the older population.

## Limitation of this study

Due to the limited content of litigation documents, we were unable to obtain more demographic data and comorbidities from older patients. Therefore, it was not possible to analyze the causes on the individual level and infer the relationship between inequalities in medical compensation and comorbidities. But this delivers opportunities for scholars to motivate medical claims equalities in the future. Additionally, medical litigation cannot cover all medical errors in practice, but the conclusions of this study still have an assertive impact on improving the health equality of older people.

## Data availability statement

The original contributions presented in the study are included in the article/Supplementary material, further inquiries can be directed to the corresponding authors.

## Author contributions

PL and YY: study concept and design, analysis, and interpretation of data. YY and JC: acquisition of subjects and data. PL, YY, and JC: preparation of the manuscript. All authors agree to be accountable for the content of the work.

## Funding

This study was supported by the Liberal Arts and Social Sciences Fund from the Ministry of Education in China (17YJA840010), Fundamental Research Funds for the Central Universities (JBK21Y53 and 19JNQM11), and 2019 Innovation Projects of General Universities in Guangdong Province (2019WTSCX002).

## Conflict of interest

The authors declare that the research was conducted in the absence of any commercial or financial relationships that could be construed as a potential conflict of interest.

## Publisher's note

All claims expressed in this article are solely those of the authors and do not necessarily represent those of their affiliated organizations, or those of the publisher, the editors and the reviewers. Any product that may be evaluated in this article, or claim that may be made by its manufacturer, is not guaranteed or endorsed by the publisher.
