# Supplementary material for: Gender differences in medical errors among older patients and inequalities in medical compensation compared with younger adults
Source: Front Public Health. 2022 Sep 20;10:883822. doi: 10.3389/fpubh.2022.883822 (PMC9540365; doi:10.3389/fpubh.2022.883822)
Supplement: Supplementary file 1 [file Table_1.DOCX]

| Class 1 error | Class 2 error | Class 3 error | Code |
| --- | --- | --- | --- |
| Errors of Medical Administration | deficiency of certification | Excessive practice in medical institution | 1101 |
|  |  | Unqualified practice of staffs | 1102 |
|  | Deficiency of resources | Deficiency of medical post setting | 1201 |
|  |  | Deficiency of human resource arrangement | 1202 |
|  |  | Defects of medical item supply | 1203 |
|  | Institutional deficiency | Deficiency of important institution | 1301 |
|  |  | Breach of important institution | 1302 |
|  |  | Laissez-faire | 1303 |
| Errors of Medical process | misidentification | Errors in identification of patients | 2101 |
|  |  | Errors in identification of operation position | 2102 |
|  |  | Error in identification of similar items | 2103 |
|  | Post inaction | AWOL (Absent Without Leave) | 2201 |
|  |  | Unusual status on duty | 2202 |
|  |  | Idle in the performance of post duties | 2203 |
|  | Errors of delivery | Critical value not reported in time | 2301 |
|  |  | Ignore important inspection results | 2302 |
|  |  | Loss of vital inspection results | 2303 |
|  |  | Loss of vital specimens | 2304 |
|  |  | Error in transmission of oral order | 2305 |
| Errors of Medical technology | Diagnosis related | Misdiagnosis | 3101 |
|  |  | Delayed diagnosis | 3102 |
|  |  | Missed diagnosis | 3103 |
|  | Examination related | Error in manipulation of inspection | 3201 |
|  |  | Errors in data report | 3202 |
|  |  | Insufficient inspection | 3203 |
|  | Therapy related | Delay of timing during treatment | 3301 |
|  |  | Errors in treatment plan | 3302 |
|  |  | Errors in manipulation during treatment | 3303 |
|  |  | Improper transfer between hospitals or departments | 3304 |
|  |  | Improper observation during treatment | 3305 |
|  | Drug related | Off-label use | 3401 |
|  |  | Ignore contraindications | 3402 |
|  |  | Errors in prescription of drug name | 3403 |
|  |  | Wrong drug dosing | 3404 |
|  |  | Errors in route of drug | 3405 |
|  |  | Contraindication of drug compatibility | 3406 |
|  |  | Errors in drug dispensing | 3407 |
|  |  | Errors in drug enforcement | 3408 |
|  |  | Defects of drug technology | 3409 |
|  |  | Errors in use of drug entrust | 3410 |
|  | Transfusion related | Delayed transfusion | 3501 |
|  |  | Unnecessary blood transfusion | 3502 |
|  |  | Errors in blood type during transfusion | 3503 |
|  |  | Infection by blood transfusion | 3504 |
|  |  | Improper disposal of transfusion reaction | 3505 |
|  | Surgical operation related | Improper comprehension of surgical indication | 3601 |
|  |  | Poor judgment of surgical contraindications | 3602 |
|  |  | Improper selection of surgical program | 3603 |
|  |  | Inadequate preoperative preparation | 3604 |
|  |  | Improper timing of surgery | 3605 |
|  |  | Improper change of surgical protocol | 3606 |
|  |  | Failed to cure the protopathy | 3607 |
|  |  | Intraoperative damage of tissues and organs | 3608 |
|  |  | Complication of post operation | 3609 |
|  |  | Improper decision-making of surgical pathology | 3610 |
|  |  | Foreign body left in body after surgery | 3611 |
|  |  | Postoperative complications were not found in time | 3612 |
|  |  | Improper treatment of postoperative complications | 3613 |
|  |  | Missed important postoperative diagnosis | 3614 |
|  |  | Non-observation for patient before surgery | 3615 |
|  |  | Preoperative misdiagnosis of disease | 3616 |
|  | Anesthesia related | Errors in choice of narcotic drugs | 3701 |
|  |  | Errors in dosage of narcotic drugs | 3702 |
|  |  | Errors in manipulation during anesthesia | 3703 |
|  |  | Neglecting the patient's condition during anesthesia | 3704 |
|  |  | Neglecting the patient's condition during the recovery phase of anesthesia | 3705 |
|  |  | Improper handling of anesthesia | 3706 |
|  | Nosocomial infection | Defect of surgical hygiene | 3801 |
|  |  | Defect of disinfection and sterilization | 3802 |
|  |  | Infection during aseptic surgery | 3803 |
|  |  | Defect in disposal of clinical waste | 3804 |
|  |  | Irrational application of antibiotics | 3805 |
|  | Nursing related | Improper monitoring of the condition | 3901 |
|  |  | Error in order of handling and execution | 3902 |
|  |  | Improper handling of general nursing | 3903 |
|  |  | Errors in nursing during invasive manipulation | 3904 |
|  |  | Errors in life nursing | 3905 |
|  |  | Improper disposal of emergency | 3906 |
| Errors of Medical product | Apparatus related | Defect in qualification of medical device | 4101 |
|  |  | Abnormal source of medical device | 4102 |
|  |  | Necessary medical device missing/not working properly | 4103 |
|  |  | Unqualified quality of medical device | 4104 |
|  |  | Improper use of medical device | 4105 |
|  |  | Expiration of medical device | 4106 |
|  |  | Product quality label not retained | 4107 |
|  | Dosage related | Defect in qualification of drug | 4201 |
|  |  | Unlawful source of drug | 4202 |
|  |  | Expiration of drug | 4203 |
|  |  | Defect in quality of drug | 4204 |
|  |  | Improper storage of drug | 4205 |
| Errors of Medical environment | Hospital facilitates | Damage caused by defects of facility quality | 5101 |
|  |  | Human damage caused by defects of facility maintenance | 5102 |
|  |  | damage caused by improper use of facilities | 5103 |
|  | Food safety | Providing quality defective food causing damage | 5201 |
|  |  | Human damage caused by supply of improper food | 5202 |
|  | Safety guarantee | Personal injury caused by incomplete security obligations | 5301 |
|  |  | Loss of property caused by incomplete security obligations | 5302 |
| Errors of Medical humanity | Doctor-patient communication | Cold and rude attitude | 6101 |
|  |  | Misunderstanding caused by miscommunication | 6102 |
|  |  | Improper promise of healing | 6103 |
|  |  | Improper disparagement of medical colleagues | 6104 |
|  | Infringement of informed consent | Not informed of important condition information | 6201 |
|  |  | Insufficient implementation of informed consent obligations | 6202 |
|  |  | Insufficient implementation of informed consent obligations of invasive treatment | 6203 |
|  |  | The actual manipulation is inconsistent with the informed consent | 6204 |
|  | Infringement of privacy | Invasion of patient privacy during medical treatment | 6301 |
|  |  | Disclosure of patient privacy information | 6302 |
|  | Overtreatment | Unnecessary inspection items | 6401 |
|  |  | Apparently unnecessary treatment | 6402 |
| Errors of Medical document | Medical record writing | Malicious falsification and manipulation of medical records | 7101 |
|  |  | Not writing medical records in time | 7102 |
|  |  | Informal writing or modification of medical records | 7103 |
|  |  | Incomplete medical records | 7104 |
|  | Medical record storage | Malicious concealment and disposal of medical records | 7201 |
|  |  | Loss of medical records | 7202 |
|  |  | Refuse to copy the sealed medical records according to law | 7203 |
